# Efficient White LEDs Using Liquid-state Magic-sized CdSe Quantum Dots

**DOI:** 10.1038/s41598-019-46581-2

**Published:** 2019-07-11

**Authors:** Sadra Sadeghi, Sirous Khabbaz Abkenar, Cleva W. Ow-Yang, Sedat Nizamoglu

**Affiliations:** 1 Graduate School of Materials Science and Engineering, Koc University, 34450 Istanbul, Turkey; 2 Department of Engineering and Natural Sciences, Sabanci University, Istanbul, 34956 Turkey; 3 Department of Biomedical Sciences and Engineering, Koc University, 34450 Istanbul, Turkey

**Keywords:** Synthesis and processing, Quantum dots

## Abstract

Magic clusters have attracted significant interest to explore the dynamics of quantum dot (QD) nucleation and growth. At the same time, CdSe magic-sized QDs reveal broadband emission in the visible wavelength region, which advantageously offer simple integration of a single-type of nanomaterial and high color rendering ability for white light-emitting diodes (LEDs). Here, we optimized the quantum yield of magic-sized CdSe QDs up to 22% via controlling the synthesis parameters without any shelling or post-treatment process and integrated them in liquid-state on blue LED to prevent the efficiency drop due to host-material effect. The fabricated white LEDs showed color-rendering index and luminous efficiency up to 89 and 11.7 lm/W, respectively.

## Introduction

**Figure 1:**
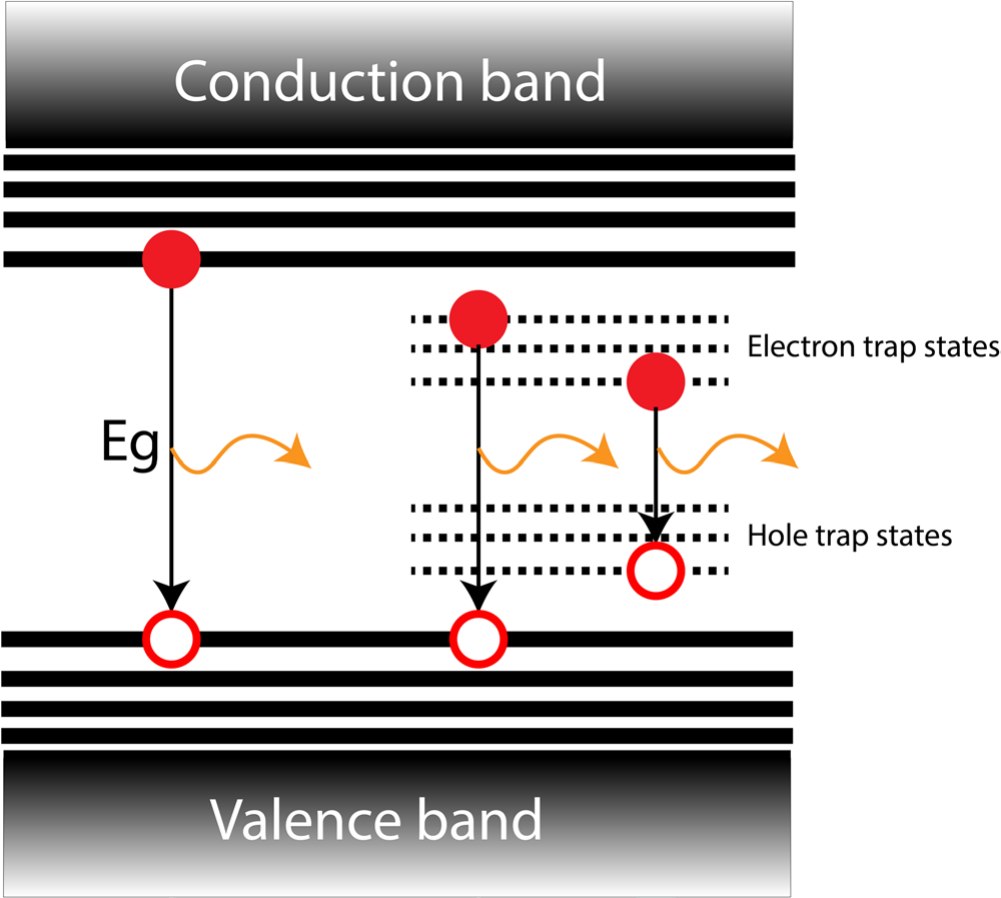
The schematic representation of energy band diagram of surface trap-states emitting QDs. The electron and hole recombinations from the trap states lead to the emission in the visible spectrum, which collectively results in white light.

Quantum dots (QDs) are formed with an evolution from molecules to crystalline nanosolids^1,2^. Among these nanosolids, magic clusters or magic-sized nanoparticles have attracted significant attention to understand the non-classical nucleation and growth of inorganic nanoparticles due to their controllable synthesis^1^. Depending on the geometry of the close-packed nanoclusters, they consist of specific number of atoms due to high thermodynamic stability^3^, and they show heterogeneous growth, which indicates a discrete jump from one structure with a specific number of atoms to another one^4^. Hence, these magic-sized QDs were applied as a seed to grow exotic structures such as nanorods, tetrapods, belts, nanoplatelets, sheets and ribbons^5–12^. The white emission from the magic-sized QDs is due to their surface trap-states. At the surface of the CdSe QDs, there are some discoordinated Se atoms, which would provide the electrons and holes trap states inside the band gap region, and can be considered as dangling bonds^13^. The range of radiative recombinations in trap-sates will collectively lead to the white emission (Fig. 1). Beside the structural properties, magic-sized QDs exhibit high surface to volume ratio, which leads to a high density of trap-states on the surface. In general, trap-states in QDs are not desirable due to the inefficiency that they lead for optoelectronic device applications^14^. Because of that, trap-states were passivated by binding a suitable ligand on the surface^15^ or by the growth of a shell^16^. Differently, the trap-states of the magic-sized QDs show broadband surface-state emission, which covers the entire visible region and can be used for light-emitting diodes (LEDs)^17–19^. Advantageously, they have benefitted a simple core structure with the integration of only one type of QDs comparing to the other traditional QD-based white LEDs, which use two or more types of QD layers^20,21^. In addition to integration, simple synthesis of these nanomaterials can enhance its use for LED application^22^. For that, magic-sized CdSe QDs are the appropriate nanomaterials that can have both simple integration and synthesis at the same time.

However, the quantum yield of magic-sized CdSe QDs are poor and white LEDs made of them have low efficiency. Schreuder and co-workers synthesized ultra-small white-emitting CdSe QDs with quantum yield of ~4%^23^. Later, they increased the quantum yield of ultra-small CdSe QDs to 45% by using formic acid for approximately two days post-treatment; even with this treatment, the predicted luminous efficiency levels of the white LEDs remained only 3.8 lm/W^24^. Other than CdSe QDs, Sapra and co-workers synthesized white-emitting CdS QDs with quantum yield of 17%^25^. Organic-capped ZnSe QDs^26,27^, and white-emitting layered perovskite with quantum yield of 9% have also been investigated^28^. Moreover, magic-sized Zn_x_Cd_13−x_Se_13_ alloyed structures with maximum quantum yield of 6% were achieved by Mn doping^29^. Recently, efficient white-emitting core/shell QDs were reported, which were prepared with a synthesis procedure that requires Mn doping on copper gallium sulfide QDs and extra step of zinc sulfide shell growth on core QDs^30^. Although there are worries about the industrial use of cadmium-based nanomaterials in devices due to their toxic material content^31^, they continue attract significant scientific attention on LEDs due to their advantageous optical properties^32–36^.

Different from the previous studies, we maximized the quantum yield of white-emitting CdSe QDs by controlling the synthesis parameters of reaction time and temperature, and their quantum yields reached up to 22% without shell formation or post treatment process for white LEDs. To prevent a possible decay of the QD efficiency in device architecture due to host material effect, we hybridized QDs in liquid-state on blue LED die that led to a white LED with a color rendering index up to 89 and a luminous efficiency of ~10 lm/W at a high current injection level of 0.1 A, which was higher than the previous studies (Table 1).

**Table 1:** Comparison between luminous efficiency of white-emitting QD-based LEDs.

Reference	Luminous efficiency
^48^	Up to 1.9 lm/W
^49^	Less than 3 lm/W
^24^	3.8 lm/W
^23^	4.83 × 10^−5^ lm/W
^50^	Less than 1 lm/W
^51^	Up to 11.93 lm/W
^30^	Up to 29.3 lm/W

## Materials and Methods

### Synthesis of white-emitting CdSe quantum dots

White-emitting CdSe QDs were synthesized based on the previous method^9,10^. In a typical synthesis, 0.4 mmol CdO (0.0515 g, >99.99% Aldrich), 0.8 mmol of octadecylphosphonic acid (0.268 g, >98% TCI) and 16 ml of tri-n-octylamine (>97% TCI) were mixed together in a 100 ml three-neck flask. The solution was heated to 150 °C under nitrogen inert atmosphere. At this temperature, the solution was evacuated by vacuum and placed under inert atmosphere repeatedly. After degassing process, the solution was heated to 310 °C under nitrogen atmosphere. Once the temperature was stable, 1 ml of 0.792 g selenium (>99.5% Aldrich) in trioctylphosphine (90% Acros) precursor (2 M TOPSe) was injected into the solution. After 180 seconds of the reaction time, the heating was stopped and the mantle was removed. The synthesized core CdSe QDs were purified, centrifuged and re-dispersed in toluene. To ensure the reproducibility and scalability of the CdSe QDs synthesis, each synthesis was performed three times (N = 3).

### Instrumentation and characterization

We carried out the UV/Visible absorption and photoluminescence spectra of QDs by Edinburgh Instruments Spectroflourometer FS5 with 150 W Xenon lamp combined with an excitation monochromator. The excitation wavelength was adjusted to 375 nm with 2 nm FWHM of band pass filter. The reported optical density, absorbance and photoluminescence spectroscopy measurement was performed by using a standard 1 cm × 1 cm quartz cuvette. A single photon counting photomultiplier tube (R928P) was used as emission detector. We measured absolute fluorescence quantum yield values by using an integrating sphere with an inner diameter of 150 mm by using FS5 system. All the quantum yields were measured in liquid-state at room temperature. We carried out time-resolved microscopy by Picoquant MicroTime 100 Time-resolved Fluorescence Microscope. A PDL 800-D diode laser driver for picosecond pulses combined with a 375 nm laser head was used as the excitation source with a repetition rate of 8 MHz. A single photon sensitive detector (PMA Hybrid 50) based on a photomultiplier tube (R10467 from Hamamatsu) was used. The time-correlated single photon counting electronics of HydraHarp 400 was adjusted to a resolution of 4 ps. The samples were measured at room temperature.

### Lens making procedure

For making semi-spherical lens, we mixed 1 g of polydimethylsiloxane (PDMS) SYLGARD 184 Elastomer with 0.1 g of SYLGARD 184 curing agent and stirred until bubbles appeared in the mixture. Then, the mixture was degassed in the vacuum desiccator for 20 minutes until the bubbles were disappeared completely. We poured the mixture into the pre-fabricated aluminum mold, and heated at 70 °C for 6 hours for completion of PDMS curing process. After heating was finished, the aluminum mold was opened and the lens was peeled off. The final product was a semi-spherical lens with outer diameter of 9 mm and inner diameter of 7 mm and the thickness of 1 mm.

### LED device making procedure

For making LED device, we mounted the blue chip on PCB board and soldered two electrical wires for connection to the voltage supply. Then, the PDMS lens was attached to the LED PCB board using NOA 68 UV curable polymer. UV curable polymer was dripped on the sides of the lens, and cured for 20 minutes with the direct exposure of 365 nm UV irradiation. This process was repeated two times to assure that the structure was leakage proof. As for close-packed state LED, UV curable polymer was mounted around the blue chip to prevent the liquid from moving to the board edge. 130 µL of white-emitting QDs solution with optical density of 0.068 (the same amounts as liquid-state QD-LED) was poured on top of blue chip to dry.

### LED measurements

For LED measurement, we used EP-B4040F-A3 InGaN/GaN 350 mA blue LED chip from Secol Company with illumination wavelength at 455 nm. We mounted the chips on a PCB board. The chips were used without any lens. We performed LED measurements with multi-port Ocean Optics integrating sphere. The detector was Ocean Optics Torus (with an optical resolution of 1.6 nm).

## Results and Discussion

**Figure 2:**
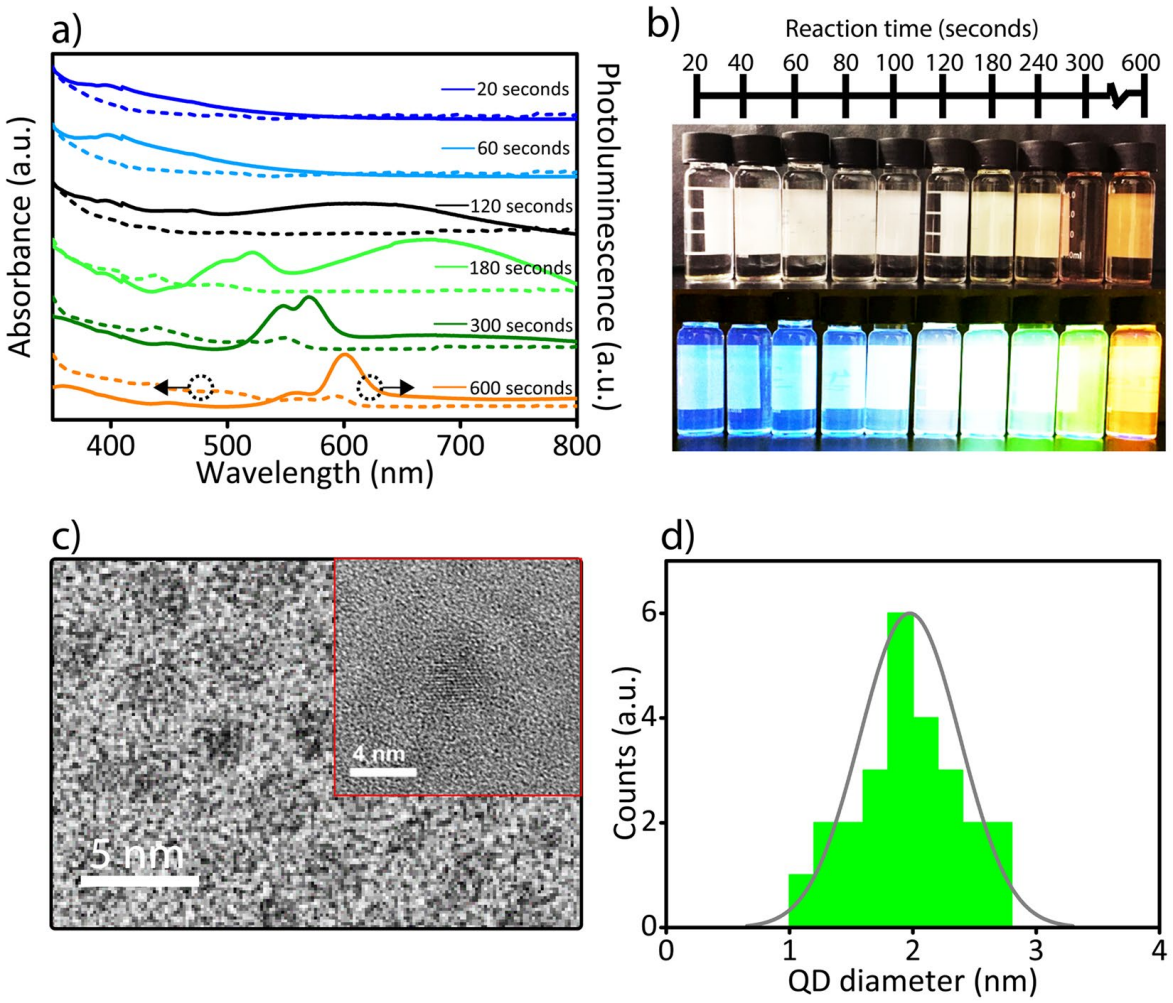
The optical properties of CdSe QDs with different reaction times. **(a)** The absorbance (dashed line) and photoluminescence (solid line) spectra of the synthesized CdSe QDs at different reaction times ranging from 20 to 600 seconds at 310 °C. (**b**) The photograph of synthesized CdSe QDs at different reaction times at 310 °C under (upper panel) ambient light and (lower panel) 365 nm UV irradiation. **(c)** TEM images of synthesized CdSe QDs at 310 °C and 180 seconds. Inset: the HRTEM image of the synthesized QDs. (**d**) The size distribution of the synthesized magic-sized CdSe QDs.

To achieve efficient and broad photoluminescence spectrum, which covers the entire visible region, we optimized radiative surface trap-states of CdSe QDs to find the point with highest quantum yield (known as photoluminescence bright point^37^). The photoluminescence bright point was demonstrated by Peng and co-workers by investigation of CdSe QDs in different reaction times, temperatures and precursor ratios. They observed that regardless of the system, solvent and the semiconductor nanocrystal size or shape, there always exist a point with highest quantum yield, which could be found by optimization of synthesis parameters^37^. To find the photoluminescence bright point in our synthesis method, we adjusted the synthesis parameters of hot injection method including reaction time and temperature. After the injection of selenium precursor into the cadmium-containing solution, the CdSe QDs started to nucleate and grow larger until the designated time. Initially, we determined a suitable reaction time for efficient broadband photoluminescence. For that, we selected a reaction temperature of 310 °C and aliquots were taken for different times after the injection of selenium precursor (Fig. 2a). During the initial times of the reaction (until 120 seconds), the formed QDs were ultra-small, which had an absorbance without any distinct narrow peak in the deep blue and UV region (Fig. 2a). In addition, these ultra-small QDs only had emission covering the deep blue and UV region, which is not suitable for white LED application. At 120 seconds, a broad tail of photoluminescence spectrum up to 800 nm was observable in the visible region. At 180 seconds, there exist two photoluminescence peaks one at 520 nm, which was possibly originated from the recombination between conduction and valence band and another one at 678 nm, which was due to the surface-states emission. In comparison with 120 seconds, the photoluminescence spectrum at 180 seconds experienced a red-shift due to size increase of the QDs. After 180 seconds, the effective trap-states emission lost its strength and the emission of the inter-band transitions became more dominant, which could be also clearly observed by their emission color detected by naked eye (Fig. 2b). At the same time, the absorption spectrum also started to reveal clear excitonic transitions. Hence, while the size of the QDs increased, the emission strength of the surface trap-states significantly decreased, which was due to the reduced surface to volume ratio and lower probability of carrier coupling to trap-states due to the larger core size^17^. To further prove the structure of the magic-sized QDs, we investigated the synthesized QDs by using HRTEM, which shows the particle size and lattice fringes (Fig. 2c-inset). The size distribution in TEM image showed the nanoparticles with the averaged diameter of 1.86 nm, which was in agreement with other magic-sized QD studies^23,24^ (Fig. 2c and 2d).

**Figure 3:**
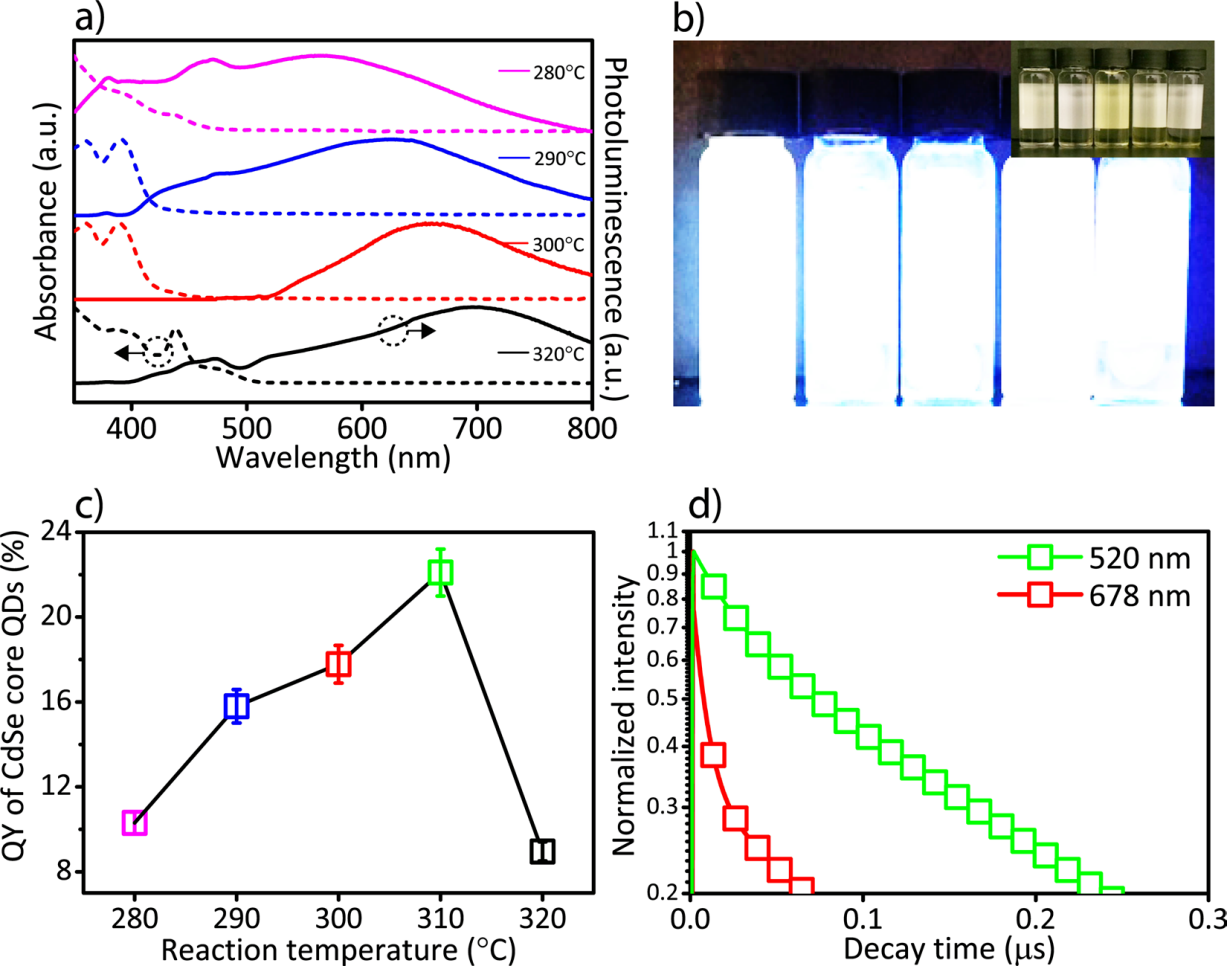
The optical properties of white-emitting CdSe QDs synthesized at different reaction temperatures at the reaction time of 180 seconds. (**a**) The absorbance (dashed line) and photoluminescence (solid line) spectra of synthesized CdSe QDs at different reaction temperatures ranging from 280 °C to 320 °C. (**b**) The photograph of synthesized CdSe QDs at different reaction temperatures under 365 nm UV irradiation. Inset: the photograph of the as-synthesized QDs under ambient light. (**c**) The quantum yield of white-emitting CdSe QDs at different reaction temperatures ranging from 280 °C to 320 °C (the averaged quantum yield was measured from total three syntheses at each reaction temperature). (**d**) The time-resolved spectra of white-emitting CdSe QDs at 520 nm (i.e., inter-band transition), and at 678 nm (i.e., surface states transition).

To optimize the efficiency of QDs, we investigated the quantum yield of the reaction temperatures between 280 °C and 320 °C at the reaction time of 180 seconds, which already showed strong trap-states emission at 310 °C (Fig. 3a). Similarly, in this reaction time interval (180 seconds), CdSe QDs were emitting white color under 365 nm UV irradiation (Fig. 3b) due to the broad photoluminescence spectra. We measured the quantum yield of the QDs (in toluene) in an integrating sphere at an excitation wavelength of 375 nm (FS5, Edinburgh Instruments). The quantum yield increased from 10.3% to 22.1%, as the reaction temperature increased from 280 °C to 310 °C and decreased to 8.9% at 320 °C (Fig. 3c). Therefore, in the following LED studies, we will use QDs with the maximum efficiency, which were synthesized at 310 °C for 180 seconds.

To investigate the dynamics of radiative recombination mechanisms related to inter-band and surface-states transitions, the time-resolved photoluminescence measurement was performed for white-emitting CdSe QDs synthesized at 310 °C at the reaction time of 180 seconds (Fig. 3d). The peak at 520 nm was attributed to inter-band recombination and the peak at 678 nm was attributed to surface state recombination (see Fig. 2a). We used two band-pass filters at the photoluminescence peak wavelengths, excited the QDs in toluene at 375 nm and PL decays were fitted by two exponential decays^38,39^. While the inter-band transition revealed considerably shorter fluorescent decay time (τ = 15 ns), the surface state emission showed longer lifetime (τ = 129 ns) due to trapped charge carriers (Fig. 3(d))^40^.

**Figure 4:**
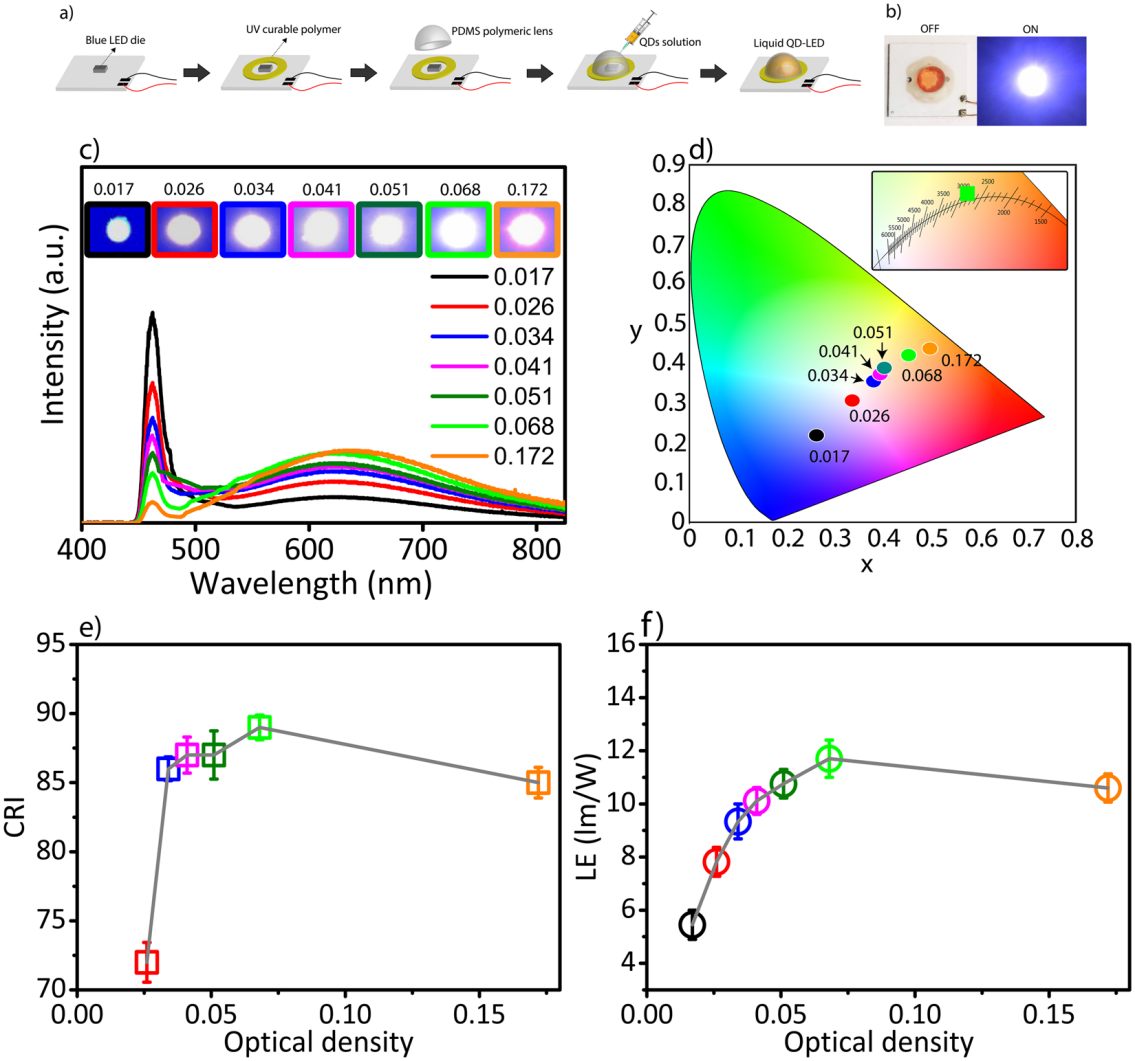
White-emitting CdSe QD-based LEDs. (**a**) The schematic of LED device fabrication. (From left to right) UV curable polymer was used to adhere the blue chip board to the PDMS polymeric lens. After curing, QD solution was injected into the polymeric lens by using a typical micro-syringe. (**b**) The photograph of liquid QD-LED (left) under the ambient light and (right) when it was illuminating with 10 mA injection current. (**c**) The LED spectra at different optical densities of white-emitting CdSe QDs ranging from 0.017 to 0.172. The blue LED die was used as pump. (**d**) (x, y) tristimulus coordinates of the white-emitting QDs at different optical densities. Inset: The zoomed area in which the green square (with optical density of 0.068) showed the highest color rendering index (89) and highest luminous efficiency (11.7 lm/W). (**e**) The color rendering index (CRI) and (**f**) the luminous efficiency (LE) of the white QD-LEDs at different optical densities ranging from 0.017 to 0.172 (The averaged CRI and LE data was measured from three LEDs).

The trap-states emission can couple to the host polymeric material in a conventional LED configuration, which can lead to additional non-radiative recombination. As a solution for surface-state emitting QDs, we integrated white-emitting CdSe QDs in liquid-state on blue LED die. For that, we fabricated a polymer lens made of PDMS and positioned it on top of the blue chip by fixing it with a UV curable resin (Fig. 4a-schematic). Then, the QD solution in toluene was injected onto the die by using a typical syringe (Fig. 4b). PDMS is a flexible polymer that has the ability to close the penetration hole after the injection^41^. This ability enabled to inject the QDs solution inside the polymeric lens without further sealing of the lens due to the self-recovering ability of the PDMS. To remove the air bubble inside the lens after the injection, another micro-syringe was used for evacuation (Fig. S1 supplementary information). The lens preparation and liquid-state integration method can be scaled up for mass production due to the low cost and high stability of PDMS polymer^41–43^. At the same time, the liquid-state integration of QDs can improve the cooling ability of the LEDs which prevents the efficiency decrease by increasing the injection current^44^. To generate high efficiency and high quality white light, it is important to integrate appropriate amounts of QDs on blue LED die. For that, we investigated the white-emitting QDs solution with optical densities of 0.017, 0.026, 0.034, 0.041, 0.051, 0.068 and 0.172, and measured the optical properties, respectively (Fig. 4c). The (x, y) tristimulus coordinates of the generated white light at different optical densities showed a wide range of color temperatures from 2428 K to 5219 K (Fig. 4d). At optical density of 0.068, the (x, y) tristimulus coordinates corresponded to (0.43, 0.42) in CIE 1931 chromaticity diagram (Fig. 4d). Color rendering index, which shows the ability of rendering true colors of the illuminated objects, is an important feature of white light. While the optical density rises, the contribution by the QDs increased until the point with maximum luminous efficiency reached^45^ and this boosted the color rendering index level up to 89 at the optical density of 0.068 (Fig. 4e). At the same time, LED with the QD optical density of 0.068 also showed a luminous efficiency level of 11.7 lm/W, which is the most efficient CdSe magic-sized QD-based white LED, according to our best of knowledge (Fig. 4f).

To compare the optical performance of the solid- and liquid-state LEDs, we fabricated another LED with the synthesized QDs in close-packed state, in which the same volume of the synthesized CdSe QDs (130 µL) was dried on top of the blue LED chip and the optical properties of the close-packed QD-LED was investigated (see methods section for the detailed explanation of the fabrication). For the close-packed QD-LED, the dried QDs on top of the blue LED chip showed aggregation and cracks in the film, which resulted in less color conversion (Fig. S2 supplementary information). This led to an undesirable (x, y) tristimulus point of (0.2, 0.13) outside the white region, and aggregation also induced a significant reduction of the efficiency with a luminous efficiency of only 3.1 lm/W.

**Figure 5:**
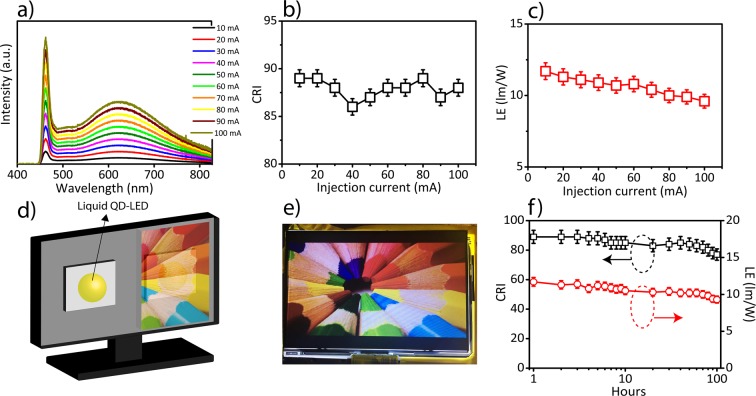
LED made of white emitting CdSe QDs (OD = 0.068) at different injection currents ranging from 10 mA to 100 mA. (**a**) The generated white light spectrum at different current levels ranging from 10 mA to 100 mA. (**b**) Color rendering index (CRI) and **(c)** the luminous efficiency (LE) at different current levels. (**d**) The schematic of 7-inch LCD display being illuminated with two magic-sized QD based white LEDs. (**e**) The photograph of an image generated by the 7-inch LCD display. (**f**) The optical stability of a magic-sized CdSe QD based white LED during 100 hours.

At higher injection currents, the LED spectra do not show a significant change (Fig. 5a) and the change in the tristimulus coordinates corresponded to Δx = 0.01 and Δy = 0.02 from 10 mA to 100 mA. As the injection current increased from 10 mA to 100 mA, color rendering index remained almost constant (varying in-between 86–89), which showed the high stability in the color rendering ability of the generated white light (Fig. 5b). Moreover, by increasing the current from 10 mA to 100 mA, the luminous efficiency of the WLEDs decreased from 11.7 lm/W to 9.6 lm/W (Fig. 5c). Furthermore, we placed two QD-based white LEDs as backlight in a 7-inch LCD display (as shown in Fig. 5d), and the produced image showed that objects and colors were easily and clearly distinguishable by using trap-state emitting QDs (Fig. 5e). Moreover, the optical stability of the fabricated QDs-based white LEDs was investigated (Fig. 5f) by constant illumination of the fabricated QD-LED up to 100 hours and the luminous efficiency showed a 20.5% decrease from 11.7 lm/W to 9.3 lm/W, respectively (Fig. 5f).

The stability of the fabricated white-emitting QD-based LEDs mainly depends on the optical stability of the blue LED chip and the synthesized QDs. To understand the origin of the efficiency drop, we initially characterized the luminous efficiency of the blue LED. For that, we fabricated LED by injecting only the solvent (toluene) inside the polymeric lens and measured the intensity of the blue LED and luminous efficiency at the same time interval (Fig. S2 supplementary information). We observed that the luminous efficiency of the blue LED chip only decreased 2% (from 15.2 lm/W to 14.9 lm/W) as the illumination time was continued until 100 hours. This proves that the majority of the 25% decrease in the luminous efficiency of the fabricated QD-LED originated from the poor stability of the QDs possibly due to accelerated photo-bleaching and irreversible photo-oxidation of trap-state assisted QDs under constant illumination in the air^46^.

**Figure 6:**
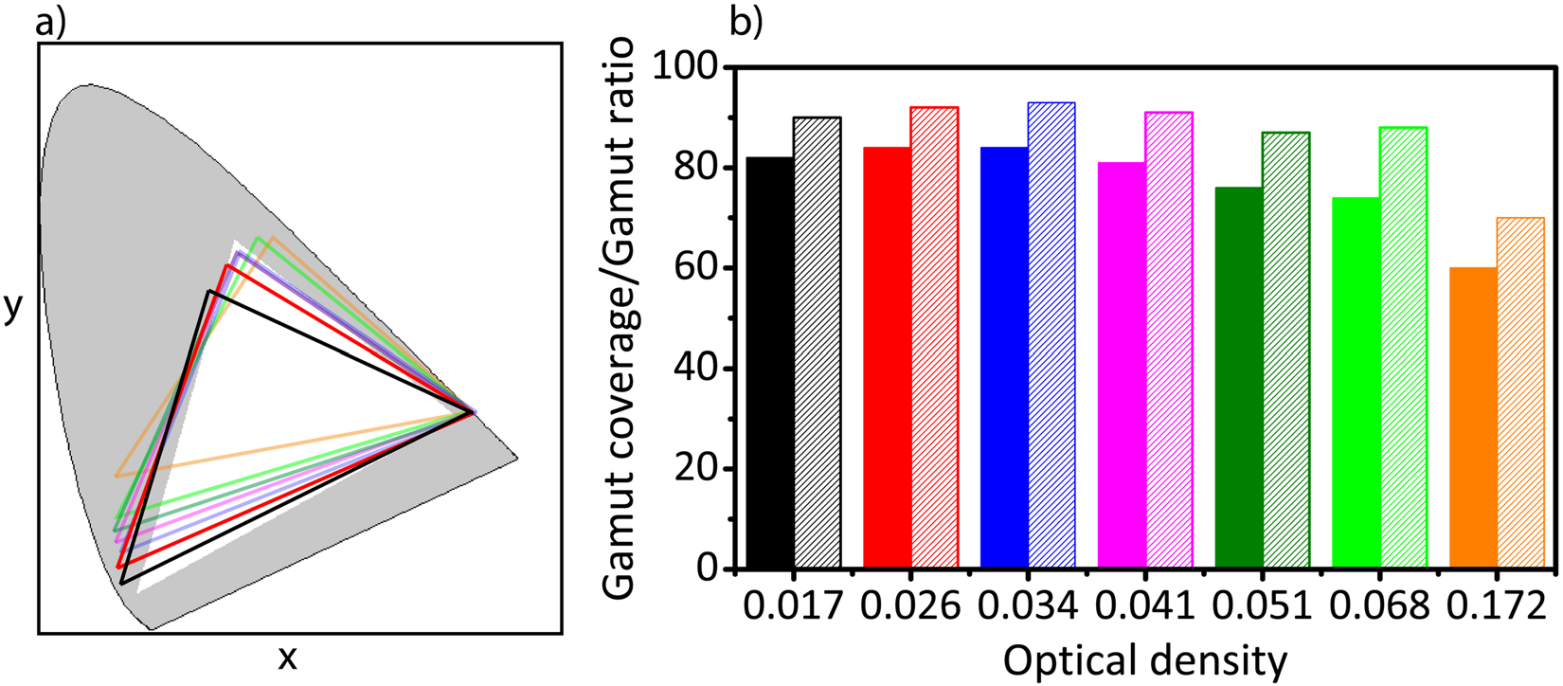
The color gamut measurements of the fabricated liquid white-emitting QD-based LEDs with different optical densities ranging from 0.017 to 0.172. (**a**) The calculated color gamut of the liquid white-emitting QD-based LEDs with different optical densities ranging from (black) 0.017, (red) 0.026, (blue) 0.034, (magenta) 0.041, (dark green) 0.051, (green) 0.068 and (orange) 0.172 in comparison with the (white filled triangle) sRGB standard color gamut in tristimulus color coordinates. (**b**) The calculated (filled bar) gamut coverage and (unfilled bar) gamut ratio based on (**a**).

The color gamuts of the fabricated liquid QD-based white LEDs with different optical densities of white-emitting CdSe QDs ranging from 0.017, 0.026, 0.034, 0.041, 0.051, 0.068 and 0.172 were calculated as shown in Fig. 6(a) and compared with sRGB standard color gamut by using blue, green and red color filters^47^ (Fig. S4 supplementary information). The calculations showed that by increasing the optical density from 0.017 to 0.034, the gamut coverage increased from 82% to 84% and gamut ratio also improved from 90% to 93%, respectively. At the same time, by further increasing the optical density to 0.172, the gamut coverage decreased to 60% and gamut ratio also dropped to 70%, respectively (Fig. 6b), which limits the applicability of the QD-LEDs with high optical densities of the white-emitting QDs for LCD displays.

## Conclusion

In this work, we synthesized white-emitting CdSe QDs with a quantum yield of 22.1% via the optimization of reaction time and temperature. The synthesis of only core QDs was performed without further shelling, doping or post-processing. We simply embedded the as-synthesized QDs in liquid-state at an optimized optical density onto a blue LED die, which simultaneously achieved a luminous efficiency of 11.7 lm/W and a color rendering index of 89 by using a single-type QDs. The integration of liquid-state magic-sized QDs suppressed the host material effect, and the broad emission spectrum of magic-sized combined with high quantum yield led to high-performance white LEDs. The liquid-state integration of white-emitting QDs with Cd-free materials can pave the way towards efficient and eco-friendly LEDs.

## Supplementary information

Supplementary Information
