# Supplementary material for: Efficient White LEDs Using Liquid-state Magic-sized CdSe Quantum Dots
Source: Sci Rep. 2019 Jul 11;9:10061. doi: 10.1038/s41598-019-46581-2 (PMC6624196; doi:10.1038/s41598-019-46581-2)
Supplement: Supplementary file 1 — Supplementary Information [file 41598_2019_46581_MOESM1_ESM.pdf]

Efficient White LEDs Using Liquid-state Magic-sized CdSe Quantum Dots

Supplementary Information

Authors

Sadra Sadeghi[1], Sirous Khabbaz Abkenar[2], Cleva W. Ow-Yang[2] and Sedat Nizamoglu[1,3,*]

Affiliation

1. Graduate School of Materials Science and Engineering, Koç University, Istanbul, 34450, Turkey.
2. Department of Engineering and Natural Sciences, Sabanci University, Istanbul, 34956, Turkey.
3. Department of Biomedical Sciences and Engineering, Koç University, Istanbul, 34450, Turkey.

* Corresponding Author: snizamoglu@ku.edu.tr

The injection procedure for the fabrication of liquid QD-LED.

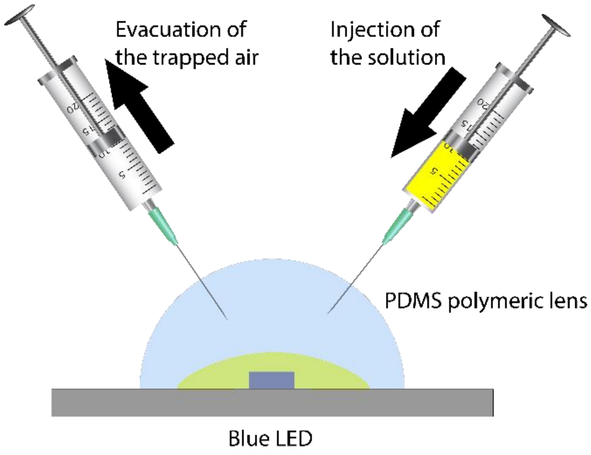

Figure S1 | The schematic of the injection procedure, in which one micro-syringe was used for the injection of the solution, while the other micro-syringe was used to evacuate the trapped air.

Comparison between luminous efficiency of liquid and close-packed white-emitting QDs-based LEDs.

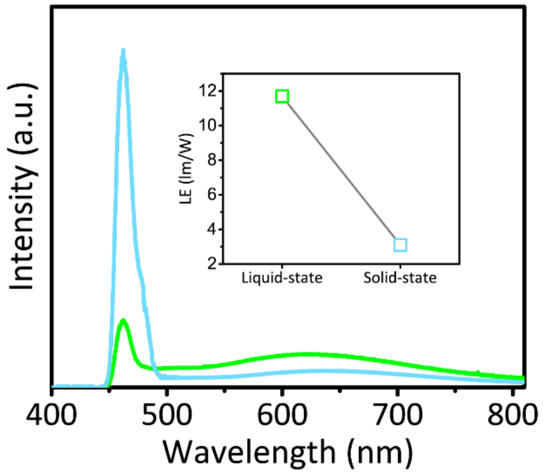

Figure S2 | The intensity spectra of the (green) liquid and close-packed (blue) white-emitting QD-based LED. Inset: the luminous efficiency of the liquid-state and close-packed solid-state QD-LEDs.

The optical properties of the blue LED.

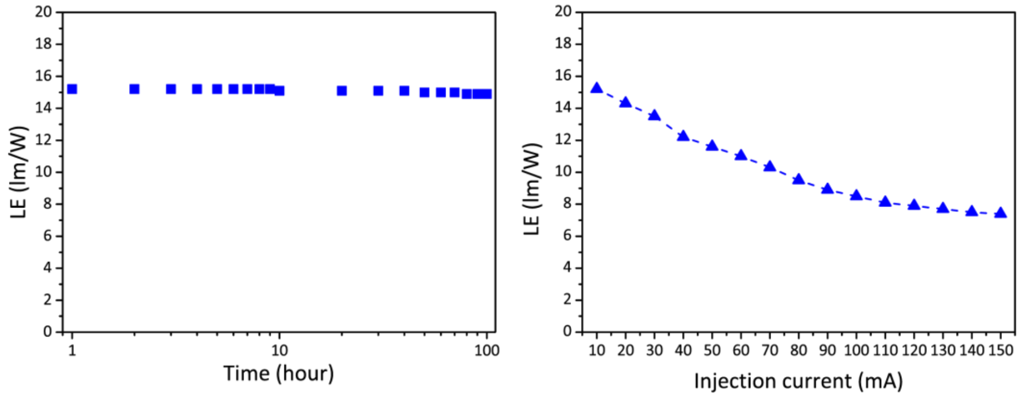

Figure S3 | The optical properties of the blue LED, which was used in this study. **(a)** The luminous efficiency of the fabricated liquid LED, which was filled only with solvent during the constant illumination time from 1 to 100 hours. **(b)** The luminous efficiency of the blue LED with different injection currents ranging from 10 mA to 150 mA.

Color filters for the color gamut calculations.

The color filters which were used to calculate the color gamut ratio were shown in Figure S2.

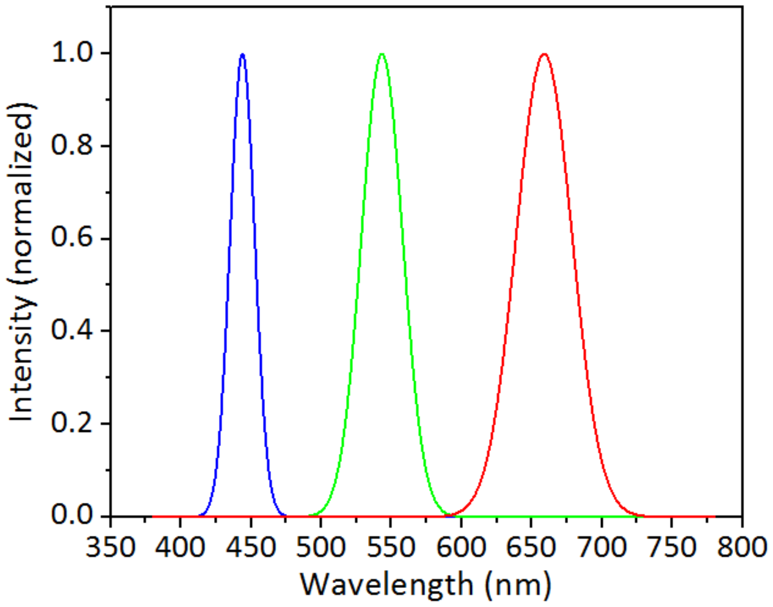

Figure S4 | The red, green and blue color filters which was used to calculate the color gamut of the generated white light.
